# Various Modalities of the Resurfacing of the Lower Limb and Its Outcome

**DOI:** 10.7759/cureus.46421

**Published:** 2023-10-03

**Authors:** Shailendra Kumar, Sudheer Kumar, Vidushi Tiwari, Neeraj Nathani, Lalmani Pal

**Affiliations:** 1 Surgery, Maharshi Vashishtha Autonomous State Medical College, Basti, Basti, IND; 2 General Surgery, Baba Raghav Das Medical College, Gorakhpur, Gorakhpur, IND; 3 General Surgery, Maharshi Vashishtha Autonomous State Medical College, Basti, Basti, IND

**Keywords:** free flap, gastrocnemius flap, open fracture, infection, limb reconstruction, muscle flap

## Abstract

Introduction

With a better understanding of local fasciocutaneous flaps, local muscle flaps, split skin grafts, myocutaneous flaps, cross-leg flaps, and microvascular free tissue transfers, soft tissue management has improved during the past few years. The present study was conducted to study the various modalities of resurfacing lower extremity wound defect and their clinical outcome in patients with lower extremities trauma.

Methodology

An observational study was done in the Department of Plastic Surgery at Baba Raghav Das (BRD) Medical College, Gorakhpur (UP), and Maharshi Vashishtha Autonomous State (MVAS) Medical College, Basti (UP), with 30 patients admitted for lower limb resurfacing irrespective of the cause of wound defect from December 2020 to November 2021. Age, comorbidities, wound features, surgical techniques, postoperative outcomes, and complications were all recorded from the patients' case sheets.

Results

All 30 patients in our study underwent some or other form of soft tissue cover suturing or healing with secondary intention or skin graft or flap cover. The majority of the patients underwent debridement and skin graft (70.0%). Flaps were used in the exposed tibia/joint/flexor surface of the limb. The donor area in all the cases was skin grafted.

Conclusion

Trauma and burns are the most common causes of soft tissue defects in the lower extremity. The major goal of the patient's treatment is to achieve rapid functional results and lesser cosmetic restoration, while using the least-invasive treatment procedure possible. The use of free flap is decreasing, while the use of local flap is increasing. However, it should be kept in mind that some procedures used to preserve function may not have the best long-term effects, and, in some instances, amputation may be required.

## Introduction

The most frequent causes of traumatic abnormalities of the leg and foot include motor vehicle accidents, falls from great heights, sports injuries, and gunshot wounds. High-energy trauma to a limb leads to massive soft tissue devitalization besides fracture of the bone [1]. Burns, especially third-degree thermal or infective wounds, also lead to skin and soft tissue loss. Thermal and infective pathology wounds are usually limited to the skin and subcutaneous tissues. While electrical (high-tension) burns are deep [2]. Regular debridement and wound preparation are dictum for further resurfacing. Systemic disease like diabetes mellitus is also responsible for limb deformity (especially foot ulcer) that needs proper hematological workup and intervention [3].

The treatment of lower extremities trauma involving bone and soft tissue damage remains difficult and fraught with danger. Teamwork between the orthopedic, medical, vascular, and plastic surgeons is necessary for successful treatment [4]. Over the past three decades, lower extremity treatment has advanced to the point where many extremities that previously required amputation are now regularly saved. High-energy trauma to a limb requires the stabilization of the fractured limb and assessment of soft tissue and vessel integrity. Grossly avulsed skin flap or muscle needs debridement [5].

A greater understanding of local fasciocutaneous flaps, local muscle flaps, split skin grafts, myocutaneous flaps, cross-leg flaps, and microvascular free tissue transfers has led to advancements in soft tissue management in recent years [6]. The method of nerve and vascular healing has been improved. Extremity salvage is a drawn-out, difficult process. Reconstructive surgery must aim to preserve a part of the body that is more useful than a prosthetic leg for an amputee [7]. As a result, patients need to be informed about the expected course and the expected functional outcome.

The present study was conducted to study the various modalities of resurfacing lower extremity wound defect and their clinical outcome in patients with lower extremities trauma.

## Materials and methods

Study design: A prospective observational study.

Study place: Plastic Surgery Department at Baba Raghav Das (BRD) Medical College, Gorakhpur (UP), and Maharshi Vashishtha Autonomous State (MVAS) Medical College, Basti (UP).

Sample size: Thirty patients admitted for lower limb resurfacing irrespective of the cause of the wound defect.

Study duration: One year (December 2020 to November 2021).

Inclusion criteria: Participants aged 14 years and older, of both genders, with skin loss in the lower extremity, able to provide informed consent for surgery, and consenting to be investigated and treated for their condition were all included in the study.

Exclusion criteria: Patients with soft tissue loss in other areas besides the lower limb like bedsores, patients with head injury or polytrauma, with uncontrolled diabetes or septicemia, patients with lower limb soft-tissue defect with associated arterial insufficiency or venous disease, and patients with lower limb soft-tissue defect with wound size very small ≤2×2 cm without any bone/tendon/joint exposure were excluded from the study.

Sampling procedure

We conducted a prospective observational study in the North Indian population with an institutional ethical clearance number BRDMC/16/CRC/2018. After admission, data regarding the study was collected by a direct interview method with the patient or their accompanying relatives, and a detailed history of age, sex, and mode of injury was collected. Thereafter, a thorough clinical examination was done, and relevant diagnostic investigations were performed by different angioimaging modalities such as ultrasonography Doppler, magnetic resonance imaging (MRI), and computed tomography (CT) were performed. Patients were thoroughly followed up in the postoperative period.

Statistical analysis

Data were recorded in a predesigned and pretested proforma. The data was coded and entered into a Microsoft Excel worksheet. The categorical data were expressed as rates, ratios, and proportions and the continuous data was expressed as mean. Analysis was done using Statistical Product and Service Solutions (SPSS) (version 23; IBM SPSS Statistics for Windows, Armonk, NY) and Microsoft Excel 2010. Percentage, mean, and frequency distribution were used in the analysis.

## Results

The present study was conducted with 30 patients to study various modalities of resurfacing lower extremity wound defect and their clinical outcome in patients with lower extremities trauma. The average follow-up of patients was four months (three months to one year).

In this study, approximately three-quarters (73.3%) of patients were male, and one-quarter (26.7%) was female with an average age of 38.3 years. Most of the patients were between the ages of 31 and 40 (Table 1).

**Table 1:** Distribution based on the sociodemographic characteristics of the patients (N=30).

Variables		Frequency (n)	Percentage (%)
Gender	Female	08	26.7
	Male	22	73.3
Age (years)	11-20	04	13.3
	21-30	04	13.3
	31-40	11	36.7
	41-50	04	13.3
	51-60	05	16.7
	15>60	02	6.7
	Mean	38.3	

**Figure 1:**
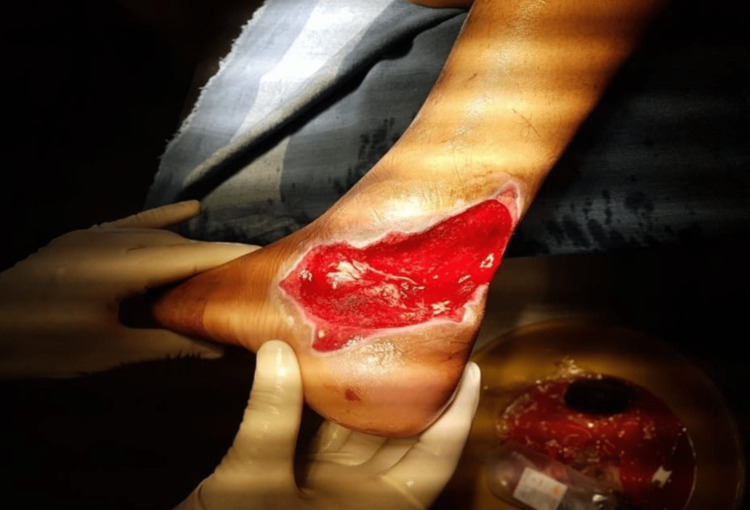
Lower limb wound (preoperative) over right ankle defect in one of the patients.

The majority of the patients were admitted with post-burn ulcers (40.0%), which include thermal burn, electric burn, etc. The duration of the wound before the resurfacing is undertaken ranged from 0 to 52 days. The majority of the wounds took 20-40 days to prepare before resurfacing. The duration of wound preparation was higher in electrical burn and infective cases. However, skin tumors required immediate cover for further intervention. A maximum number of patients had a wound over the leg (86.7%), and the other less-common places were the foot (3.3%) and ankle (3.3%) (Table 2, Figure 1).

**Table 2:** Distribution based on the etiology, duration of wound preparation, and site of the limb defect (N=30).

Variables		Frequency (n)	Percentage (%)
Etiology	Trauma	08	26.7
	Post-burn ulcer (thermal, electric, and others)	12	40.0
	Post cellulitis ulcer	09	30.0
	Skin tumor	01	03.3
Duration of wound preparation	<20 days	09	30.0
	20-40 days	14	46.7
	>40 days	07	23.3
Site of defect	Thigh and leg	02	6.7
	Leg	26	86.7
	Ankle	01	3.3
	Leg and foot	01	3.3

**Figure 2:**
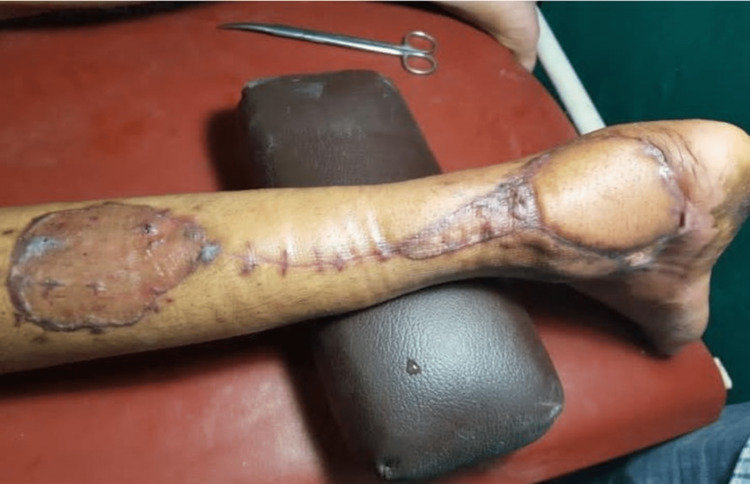
Reverse sural flap (postoperative) to cover right ankle defect in one of the patients.

All 30 patients in our study underwent some or other form of soft tissue cover- suturing or healing with secondary intention or skin graft or flap cover. The majority of the patients underwent debridement and skin grafts (70.0%). Flaps were used in the exposed tibia/joint/flexor surface of the limb. The donor area in all the cases was skin grafted. The range of wound healing was variable. It was shorter in flap cover cases (19 days) and the longest (62 days) in skin grafts. Wound healing was long in infective cases. The average duration of wound healing was 36.26 days (Table 3, Figure 2).

**Table 3:** Distribution based on the treatment modalities used (N=30).

Variables		Frequency (n)	Percentage (%)
Technique of defect closure	Debridement and skin graft	21	70.0
	Flap cover and skin graft	09	30.0
Flap used (n=9)	Fasciocutaneous flap	05	16.7
	Reverse sural flap	02	6.7
	Myocutaneous flap	02	6.7
Duration of wound healing	<1 month	12	40.0
	1 month to <2 months	17	56.7
	2 months or more	1	3.3
	Mean	36.3 days	

The postoperative course of the wound healing had some complications (early and delayed). Out of 30 cases undertaken for the study, half (50%) of the cases had no postoperative complications. These cases healed smoothly in due course of time. Twenty percent of them had suture dehiscence of the flap margin (Table 4).

**Table 4:** Distribution based on complications (early and late) observed in wound cover by different techniques (N=30).

Variables	Frequency (n)	Percentage (%)
No complication	15	50.0
Graft loss due to shear force	05	16.7
Suture dehiscence of flap margin	06	20.0
Infection leading to patchy skin graft loss	01	3.3
Delayed wound healing due to nutritional deficiency	01	3.3
Hematoma leading to patchy skin graft loss	01	3.3
Partial flap necrosis	01	3.3

Outcome of soft tissue cover in a follow-up

In the follow-up, for all the cases of patchy graft loss, suture dehiscence, or partial flap necrosis, no secondary procedure was done. The remaining wound healed with secondary intention. In the case of partial flap necrosis, the dead skin was debrided, and a new wound healed with dressings and secondary intention. One case was diagnosed with nutritional deficiency in the postoperative period, deficiency was corrected, and wound was healed with dressing. The resurfaced wound healed smoothly in the specified time. The patient became ambulatory and went back to their work in all the cases in the follow-up. None of them had any complaints except pedal edema. Dependent edema was corrected with lifestyle modification (i.e., wearing stockings while standing or walking and keeping the feet elevated over some support during rest). The overall survival rate for flap without any complication in our study was 7 out of 9. In the case of skin graft, the graft taken rate without complication was 57.24%.

## Discussion

The present study was conducted in the Department of General Surgery, BRD Medical College, Gorakhpur, from December 2020 to November 2021 on 30 patients of lower extremity wounds of various etiologies.

The patients in our study ranged in age from 14 to 66 years, with a male preponderance (male:female=2.75:1). The average age was 38.3 years. Most of the patients, between the ages of 31 and 40, showed wound affliction in this age group due to their outdoor activity, hence becoming more prone to injury. Martinov et al. in their study showed that the average age was 49.7 years [8].

In our study, post-burns (40%) and trauma (26%) are the two most common causes of soft tissue defects in the lower extremities that do not heal spontaneously because of substantial tissue loss or diminished vascular supply. Postinfection wounds, which have poor granulation and local infection, are the third most common cause. Martinov et al. in their study showed that out of nineteen, fourteen were traumatic [8]. In 8 patients (42.1%), high-energy trauma from a car accident resulted in a soft tissue defect. Another 6 faults (31.6%) were brought on by falling from a height of less than 6 m. After surgery, a defect appeared in 5 cases (26.3%). 

The primary goal of lower extremity reconstruction is to cover the wound and maintain its function. In our study, burns are the major cause of soft tissue loss in the lower extremities. Adults in our society are responsible for cooking food for the family or outside in hotels/restaurants. Workplace accidents because of carelessness or because of loosened clothes (saree and lungi) catching fire leading to third-degree burns of the lower extremity [9]. A third-degree burn needs resurfacing with a skin graft or flap reconstruction to retain the mobility of the limb. Even a second-degree burn, if neglected, is converted into a third-degree burn because of persistent infection. Hence, proper and regular dressing has to be done and infection has to be eliminated. Usually, superficial burns heal within 3 weeks. If the burn wound exceeds this period, then resurfacing by skin grafts (if healthy granulation is present) needs to be done [10].

In the present study, a maximum number of patients had a wound over the leg (86.7%), and the other less common places were the foot (3.3%) and ankle (3.3%). Martinov et al., in their study, showed that the lower third of the lower leg (52.6%) was involved in the majority of the patients followed by the site of insertion of the Achilles tendon (21.1%), distal thigh was involved in 10.5%, and plantar surface of the midfoot was involved in 10.5% [8].

In our study, the most prevalent method for covering the defect area was skin graft (70 percent). In 30% of the cases (n=09), we use a regional flap with a skin graft. In these nine patients, we used a fasciocutaneous flap and a myocutaneous flap to cover the superior or middle 1/3 or both defects. Conversely, a reverse sural flap was exclusively used for the lower 1/3 tibia or ankle or heel region. Among the flap surgeries, the fasciocutaneous flap (n=5) was the most commonly employed flap in our study to cover the defect. While myocutaneous and reverse sural flaps were used in two cases each for cover.

In this study, skin graft reconstruction was overwhelmingly preferred for abnormalities on the dorsum of the foot, proximal 2/3 of the leg, and thighs. In lower extremity repair, local flaps that are intended as fasciocutaneous are crucial as they can be applied locally to minor bone-exposed lesions without impairing muscle function. Therefore, in our study, lower extremity reconstruction was performed using five out of nine local random fasciocutaneous flaps.

Muscle function may be lost or diminished as a result of musculocutaneous flaps. Massive or atypical skin or soft tissue abnormalities can be accommodated by free flaps, but the design of a pedicled flap is typically constrained by the local anatomy, the availability of skin, and the orientation of the wound [11]. Local flaps may leave a noticeable visual flaw at the donor site that may be challenging to conceal [12]. Because of its simplicity, adaptability, low cost, and low donor site morbidity, the distally based sural nerve flap is often employed for the reconstruction of the distal third of the leg, ankle, and heel. The distal marginal necrosis of the flap typically happens when the soft-tissue defect is placed at the dorsum of the metatarsophalangeal joint, which is the exact region of the flap that one needs the most. This is true even though the sural flap has a high success rate if the peroneal artery is patent [13]. For individuals with serious lower extremity injuries, free flaps would be the best option. However, there are several relative contraindications to free flap surgery, such as electrical burns, single vessel limbs, delayed referral, and patients who have received radiotherapy after having a bone tumor removed. Patients with severe lower extremity injuries, axial vascular damage, a history of prior trauma, and blood vessel thrombosis cannot employ a free flap [14].

The present study revealed that the postoperative course of wound healing had some complications (early and delayed) in half of the patients. The majority of them (20.0%) had suture dehiscence of the flap margin. For the distal third of leg deformities, a total of two reverse flow sural flaps from the same leg were employed. In one instance, partial flap necrosis manifested at the distal end. No patient had any problems from the donor location, as demonstrated by Martinov et al. in their study [8]. In the three cases, the flap's complications were observed. Only one patient needed additional surgery (debridement and split-thickness skin grafting). In their investigation, Ong et al. documented 10 double flaps in ten individuals [15]. The typical follow-up time was 12 months. All flaps underwent initial healing without any significant issues. Flap tip necrosis and superficial infection were two instances of mild complications.

In the follow-up, for all the cases of patchy graft loss, suture dehiscence, or partial flap necrosis, no secondary procedure was done. The remaining wound healed with secondary intention. The resurfaced wound healed smoothly in the specified time. The patient became ambulatory and went back to their work in all the cases in the follow-up. The overall survival rate for flap without any complication in our study was seven out of nine. In the case of skin graft, the graft take rate without complication was 57.24%. In their investigation, Ong et al. documented 10 double flaps in ten individuals. At the end of the treatment, nine patients were ambulatory without assistance [15].

The small sample size in our study was one of its limitations. A bigger sample size might reveal possible interactions between the variables that can influence the study results. The clinical outcome after skin grafting or flap reconstructing surgery is good if it is done in time after an adequate number of patients and wound preparation. The success of flap surgery depends upon its availability in nearby of the defect; it should not be short enough to cover the defect, while transposition of flap venous insufficiency has to be taken care of. Thus, resurfacing techniques are needed to preserve the basic function of the limb while healing wounds in a timely and abnormal fashion.

## Conclusions

Trauma and burns are the most common cause of soft tissue defects in the lower extremity. The major goal of the patient's treatment is to achieve rapid functional results and lesser cosmetic restoration while using the least invasive treatment procedure possible. The location and size of the soft tissue defect, the presence of exposed significant anatomic structures, the vitality of the surrounding tissue, the patient's general health and expectations, and the existence of exposed significant anatomic structures all affect the treatments utilized to rebuild soft tissue defects. In other words, local flap usage is rising while free flap usage is declining. The use of treatments to preserve function may not, however, yield the best long-term outcomes; in some instances, amputation may be required.

## References

[1] Hoyt BW, Pavey GJ, Pasquina PF, Potter BK (2015). Rehabilitation of lower extremity trauma: a review of principles and military perspective on future directions. Curr Trauma Rep.

[2] Tiwari VK (2012). Burn wound: how it differs from other wounds?. Indian J Plast Surg.

[3] Edwards J, Stapley S (2010). Debridement of diabetic foot ulcers. Cochrane Database Syst Rev.

[4] Wen G, Zhou R, Wang Y, Lu S, Chai Y, Yang H (2019). Management of post-traumatic long bone defects: a comparative study based on long-term results. Injury.

[5] Sabapathy SR, Venkatramani H, Mohan M (2019). Initial assessment, debridement, and decision making in the salvage of severely injured lower extremity. Indian J Plast Surg.

[6] Mégevand V, Suva D, Mohamad M, Hannouche D, Kalbermatten DF, Oranges CM (2022). Muscle vs. fasciocutaneous microvascular free flaps for lower limb reconstruction: a meta-analysis of comparative studies. J Clin Med.

[7] Qureshi MK, Ghaffar A, Tak S, Khaled A (2020). Limb salvage versus amputation: a review of the current evidence. Cureus.

[8] Martinov M, Argirova M (2022). Fasciocutaneous flaps in the lower limb soft tissue reconstruction - a surgical case series. Orthoplastic Surgery.

[9] Haruyama Y, Matsuzuki H, Tomita S (2014). Burn and cut injuries related to job stress among kitchen workers in Japan. Ind Health.

[10] (2023). Treatment of superficial burns requiring hospital admission. https://www.uptodate.com/contents/treatment-of-superficial-burns-requiring-hospital-admission/print.

[11] Deramo P, Rose J (2023). Flaps: muscle and musculocutaneous. StatPearls.

[12] Rao JK, Shende KS (2016). Overview of local flaps of the face for reconstruction of cutaneous malignancies: single institutional experience of seventy cases. J Cutan Aesthet Surg.

[13] Zheng H, Liu J, Dai X, Schilling AF (2016). The distally based sural flap for the reconstruction of ankle and foot defects in pediatric patients. Ann Plast Surg.

[14] (2023). Free Tissue Transfer Flaps. https://emedicine.medscape.com/article/1284841-overview.

[15] Ong SW, Gan LP, Chia DS (2020). The double muscle gastrocnemius-soleus flap in resurfacing large lower limb defects: modifications and outcomes. J Orthop.

